# Implementation of Predictive Algorithms for the Study of the Endarterectomy LOS

**DOI:** 10.3390/bioengineering9100546

**Published:** 2022-10-12

**Authors:** Teresa Angela Trunfio, Anna Borrelli, Giovanni Improta

**Affiliations:** 1 Department of Advanced Biomedical Sciences, University of Naples “Federico II”, 80131 Naples, Italy; 2 “San Giovanni di Dio e Ruggi d’Aragona” University Hospital, 84121 Salerno, Italy; 3 Department of Public Health, University of Naples “Federico II”, 80131 Naples, Italy; 4 Interdepartmental Center for Research in Healthcare Management and Innovation in Healthcare (CIRMIS), University of Naples “Federico II”, 80131 Naples, Italy

**Keywords:** length of stay, endarterectomy, machine learning

## Abstract

Background: In recent years, the length of hospital stay (LOS) following endarterectomy has decreased significantly from 4 days to 1 day. LOS is influenced by several common complications and factors that can adversely affect the patient’s health and may vary from one healthcare facility to another. The aim of this work is to develop a forecasting model of the LOS value to investigate the main factors affecting LOS in order to save healthcare cost and improve management. Methods: We used different regression and machine learning models to predict the LOS value based on the clinical and organizational data of patients undergoing endarterectomy. Data were obtained from the discharge forms of the “San Giovanni di Dio e Ruggi d’Aragona” University Hospital (Salerno, Italy). R^2^ goodness of fit and the results in terms of accuracy, precision, recall and F1-score were used to compare the performance of various algorithms. Results: Before implementing the models, the preliminary correlation study showed that LOS was more dependent on the type of endarterectomy performed. Among the regression algorithms, the best was the multiple linear regression model with an R^2^ value of 0.854, while among the classification algorithms for LOS divided into classes, the best was decision tree, with an accuracy of 80%. The best performance was obtained in the third class, which identifies patients with prolonged LOS, with a precision of 95%. Among the independent variables, the most influential on LOS was type of endarterectomy, followed by diabetes and kidney disorders. Conclusion: The resulting forecast model demonstrates its effectiveness in predicting the value of LOS that could be used to improve the endarterectomy surgery planning.

## 1. Introduction

In recent years, public healthcare spending in Italy has increased significantly, reaching EUR 117 billion in 2019 [1]. For this reason, healthcare facilities have reduced hospital costs, which to date account for one-third of healthcare costs. Today, cost-effectiveness indicators play a significant role in the management and organization of care programs [2]. For process optimization, several techniques already popular in other settings have been implemented in healthcare sector [3,4,5,6]. Patient length of stay (LOS) is a significant factor contributing to healthcare costs; in fact, a short LOS is directly related to reduced costs [7].

The literature reports that the evaluation of LOS through advanced analytical techniques and artificial intelligence algorithms is the subject of numerous studies [8,9,10,11,12]. History has verified that some populations with high-grade carotid stenosis are at high risk of subsequent stroke [13]. Currently, vascular surgeons are aware of the influence of surgical planning on resource and cost due to the growing focus on the efficiency of medical procedures [14].

Different listed research studies [15,16,17,18] have validated the ability of endarterectomy to prevent stroke in both symptomatic and asymptomatic patients. As its efficacy in stroke prevention has been demonstrated, the number of such procedures could increase dramatically [19], where a standardized procedure concerns the removal of the accumulation of atheromatous plaque from the walls of an artery to reduce the long-term risk of stroke [16]. However, economic evaluations of this procedure, particularly of the postoperative phase, have not yet been fully addressed [20,21] and require further investigation into the factors that influence LOS after endarterectomy surgery [22]. In fact, a postoperative LOS of 2–4 days or more is generally associated with particular complications caused by the patient’s health condition; although the obvious drawback is that a 1-day LOS is often not achieved. Patients with complications cause financial loss to healthcare facilities due to the longer post-operative time and the greater healthcare expenditure [23]. Understanding the regulatory and control variables that influence the duration of postoperative LOS can be a strategy to facilitate the reduction of healthcare costs, although they vary according to the patient’s age, sex and comorbidities [21]. Identifying the factors that cause prolonged LOS is essential to improve the patient’s condition and reduce healthcare costs [22,24,25]. Several studies report advanced processing of cardiac data for diagnostic purposes [26,27,28,29,30,31] or to support the monitoring process [32,33]. The aim of the present work is to determine the factors associated with prolonged hospitalization following endarterectomy, using the clinical and organizational data collected at the “San Giovanni di Dio e Ruggi d’Aragona” University Hospital. In this study, we design a machine learning (ML)-based model for predicting LOS with the purpose of optimizing the LOS of patients undergoing endarterectomy. In addition, we evaluate and compare the effectiveness of different ML models in terms of different measures (e.g., R^2^, accuracy, precision, F-measure) to validate or reject the results obtained in our previous work [34], which focused on a subset of ML models for a limited number of years and variables without implementing any optimization process or studying the impact that selected independent variables have on LOS. With this study, it is possible to both understand the risk factors for prolonged LOS and build models that can predict these cases.

## 2. Materials and Methods

The Complex Operative Unit (C.O.U.) of Cardiology of the “San Giovanni di Dio e Ruggi D’Aragona” University Hospital made it possible to carry out this study by providing the requested data. Specifically, the dataset was extracted from the hospital’s information system and contains 2243 records regarding patients who underwent endarterectomy surgery (ICD-9 codes equal to 38.1x) from 2010 to 2020. The information collected for each patient was: gender, age, main and secondary diagnoses, year of discharge, date of admission, date of discharge and date of surgical treatment. The dataset was prepared to make it compatible with the processing of ML algorithms. Subsequent regression and classification analysis was performed by considering the following as variables:

- Gender (male/female);
- Age;
- Hypertension (yes/no);
- Diabetes (yes/no);
- Previous heart attack (yes/no);
- Embolism (yes/no);
- Hyperlipidaemia (yes/no);
- Respiratory system disorders (yes/no);
- Obesity (yes/no);
- Kidney disorders (yes/no);
- Cardiomyopathy (yes/no);
- Rhythm abnormalities (yes/no);
- Anemia (yes/no);
- Personal history of allergies (yes/no);
- Pre-operative LOS;
- Type of endarterectomy (Indicates on which vessels the endarterectomy was performed: 1, vessels of the head and neck; 2, upper limb vessels; 3, aorta; and 4, lower limb vessels).

**Figure 1:**
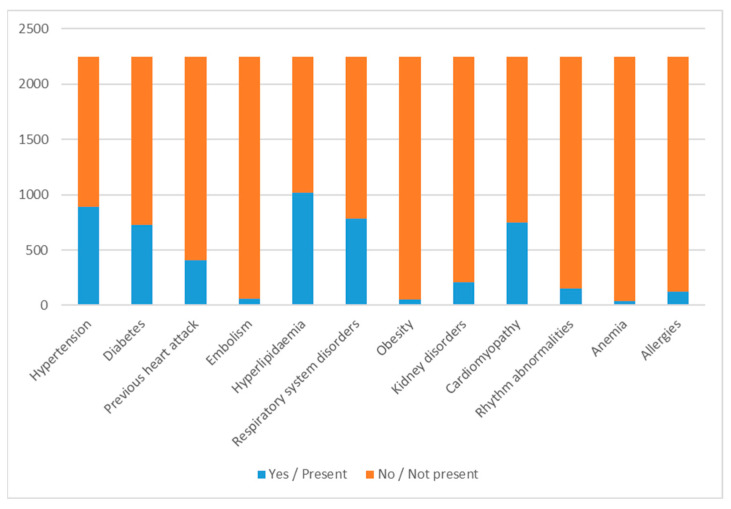
Distribution of dichotomous features in the dataset. For each comorbidity considered, the graph shows, out of the total number of subjects included in the study, the portion of patients with that comorbidity in blue, and the portion of patients without that comorbidity in orange.

With this information, the regression and classification algorithms were applied to predict the total LOS. Figure 1 shows the distribution of the dichotomous variables, where 1/Yes indicates that comorbidity is present between the patient’s primary and secondary diagnoses while 0/No indicates that the patient has no such disorder.

For the variable year of discharge, the number of discharges for each year is shown in Table 1.

From the data shown in Table 1, it can be seen that with the exception of the year 2010, where the low number of cases was due to the initial adoption of the software, the lowest number of discharges occurred in 2020 due to the spread of the COVID-19 pandemic. Finally, the distribution according to the variable type of endarterectomy is shown in Table 2.

Table 2 shows that the most performed procedure was the procedure involving the head and neck vessels, followed by the procedure involving the lower limbs.

**Figure 2:**
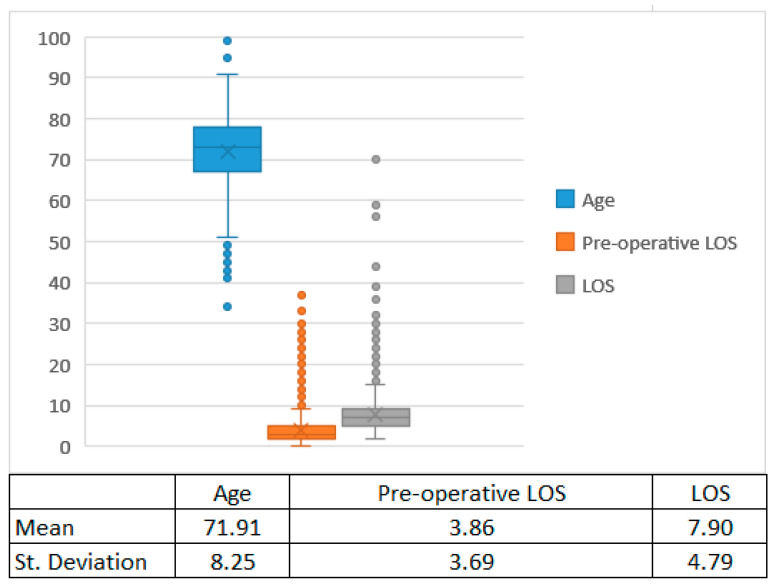
Box plots for continuous variables. The graph shows, for the continuous variables included in the study (age, pre-operative LOS and LOS), the median of the data (the middle line in the box), the mean of the distribution (the x in the box) the 25th and 75th quantiles (the bottom and top of the box), the interquartile range (IQR) (length of the box), the expected 1.5-fold variation in the data from IQR from the top and bottom (the lines extending from the box) and the outliers. The table below briefly shows the value of mean and standard deviation of the distribution of each variable.

For continuous variables, the box plots are shown in Figure 2.

Google Colaboratory (Colab) Cloud Platform was used to implement regression and ML algorithms.

### 2.1. Regression and Machine Learning Algorithms

The purpose of this section is to study different regression and classification models to predict LOS value. Gender, age, hypertension, diabetes, previous heart attack, embolism, hyperlipidaemia, respiratory system disorders, obesity, kidney disorders, cardiomyopathy, rhythm abnormalities, anemia, allergies, pre-operative LOS and type of endarterectomy were used as input variables for the algorithms to predict the subjects’ LOS. Random forest (RF), multilayer perceptron (MLP), naïve Bayes (NB), support vector machine (SVM) and decision tree (DT) were the five different classification methods used. In particular, we chose these models because they are the most widely used in ML benchmark designs [35]. Next, the regression algorithms were implemented. In addition to multiple linear regression (MLR), random forest (RF) and decision tree (DT) were also used as regression algorithms.

The choice of these methods was motivated primarily by the desire to improve code quality and the performance of learning operations on the dataset. The classifiers used were all from the scikit-learn library, which is a ML library. Data mining methods available with significantly different architectures were chosen that allowed for a tuning operation of the parameters of the classifiers.

The dataset was randomly divided into two sections to assess the goodness of models and the accuracy value achieved, with the training data collecting 80% of the total data and the test data collecting the remaining 20%. The training phase was performed on the training dataset, while the testing phase was performed on the test set. Each model assigned a value to each input sample based on the pattern learned during the training phase.

### 2.2. Parameter Optimization and Cross-Validation for Classification Algorithms

The careful adjustment of parameters was made according to the individual properties of each classifier and the goodness of fit of the resulting model was evaluated. Based on its characteristics, each algorithm had appropriate parameters to be set. The infrastructure provided by scikit-learn was used to improve hyperparameters of the algorithms.

GridSearchCV was supported to determine the best model or parameters for a specific task. In particular, the estimator and the param_grid consisting of the name of the specific hyperparameter for that estimator, and the range of values within which it should be varied, were given as the input. Table 3 shows the arbitrarily selected values for each algorithm.

The exact distribution of the dataset between training and test data could influence the accuracy value reached by each classifier. The value recorded may have been determined by chance and thus is not indicative of the model’s level of quality. To ensure that the accuracy value was not erratic, but rather the accuracy value reached by the classifier, ten-fold cross-validation was used.

To begin, a single data pair (training, test) was constructed, divided into two parts using a training ratio parameter, and the classifier was applied. The dataset was then separated into ten folds using the CrossValidator tool, which were used as independent datasets for training and testing (cv = 10 partitions of data equal in size to 10 instances of learning, using 9 for training and 1 for testing). CrossValidator calculates the average evaluation metric for the models built by fitting the estimator to the 10 pairs of separate datasets (training, test) to evaluate a particular set of parameters. CrossValidator finally refits the estimator using the best set of parameters and the entire dataset to obtain the best output.

### 2.3. Voting Technique

Each classifier has a higher level of accuracy in discriminating LOS than the others. Therefore, once the five classifiers had issued their predictions, a voting classifier (VC) used them to determine the majority class to assign to the tuple. For best results, the VC employed an ensemble technique based on majority policy. Once the predictions of the five classifiers have been gathered, the VC must use them to determine the majority class to assign to the tuple. Indeed, the VC made a prediction relating to the option that received more than half of the votes, assigning each sample the value expected by at least three of the classifiers.

There are two different types of VCs. Hard VCs classify input data according to the mode of all predictions by various classifiers, while soft VCs rank them according to probability. For the hard type, in determining the majority vote, it is possible to use constant weights or to assign different weights to the various classifiers. One way to determine these weights is to use the target metric, which in this case was accuracy.

## 3. Results

**Figure 3:**
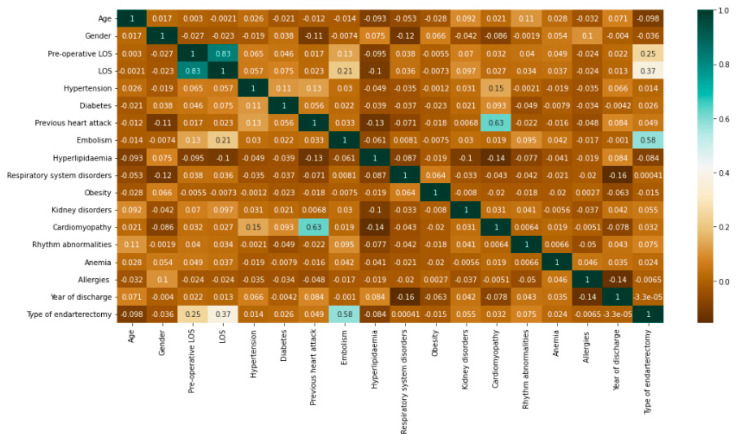
Correlation study. The diagram shows for each variable (row) the Pearson correlation value relative to all other variables included in the study (columns). In addition to the result obtained, each cell takes on a color ranging from brown (0) to dark green (1) depending on the correlation value returned, following the color gradations reported by the colored bar to the right of the chart.

Before implementing the algorithms, a correlation study was carried out to investigate the relationship between the dependent and independent variables included in the dataset. Using the Python Data Analysis Library “Pandas,” Pearson’s correlation was implemented to calculate the pairwise correlation of columns, excluding NA/null values, of all variables presented. The result is shown in Figure 3.

Among the variables, with the exception of pre-operative LOS included in LOS by definition, it was the type of endarterectomy that had the highest correlation with LOS. The highest correlation coefficient of 0.63 was recorded between cardiomyopathy and previous heart attack.

The purpose of this paper is to identify the regression and classification algorithm to predict total LOS by achieving better results. First, regression models were implemented. Table 4 shows the performance of each model.

Among the algorithms, the best was the MLR model, with an R^2^ value greater than 0.8. Table 5 shows the parameters of the MLR model obtained using IBM SPSS Statistical Software v. 27.

**Figure 4:**
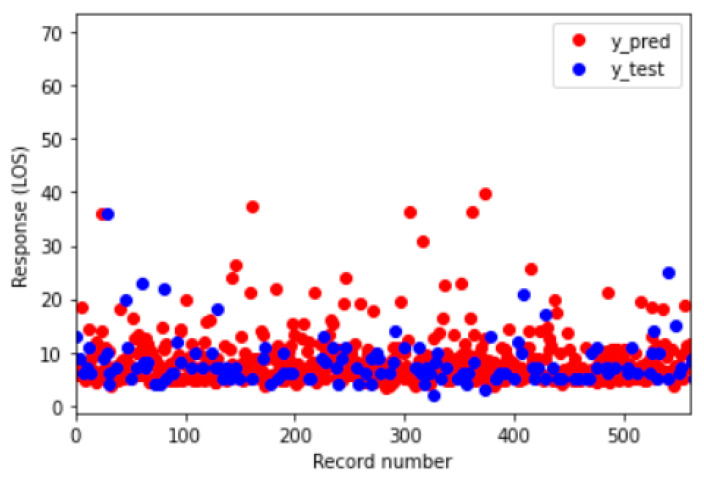
MLR output –Real value (blue) vs. prediction (red) for each observation in the test set.

Among the coefficients, the highest positive value was associated with the type of endarterectomy, followed by diabetes and kidney disorders. Finally, Figure 4 shows the difference, in graphical form, between the prediction (in red) and the actual value (in blue).

Next, the classification algorithms were implemented. To do this, the initially continuous LOS variable was divided into three groups as below:

- Group 0: LOS ≤ 5;
- Group 1: 5 < LOS ≤ 7;
- Group 2: LOS > 7.

These values were derived in order to divide the dataset equally and facilitate the classification process. The baseline characteristics of the three groups are shown in Table 6.

The table shows that Group 2 was consisted of patients with diabetes, embolism and rhythm alteration, and most of those undergoing endarterectomy on lower extremity vessels.

We then proceeded to implement the classification algorithms. The accuracy of each algorithm is after cross-validation. In ML, accuracy is defined as the ratio of correct predictions to the number of data in the test set. These values, with the addition of the optimal parameters, are shown in Table 7.

Among the algorithms, the best performance was obtained with DT. Table 8 shows the complete metrics of this algorithm.

**Figure 5:**
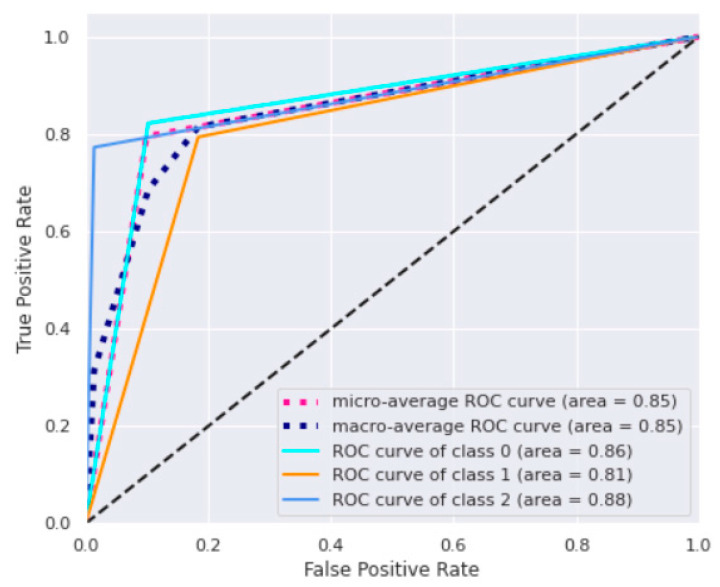
ROC curves. Figure shows: the ROC curve for each class, the micro and macro average ROC curves, and the “no benefit” line (black discontinuous line) representing a causal classifier with area = 0.5.

Precision is the ratio of correct predictions to total predictions for a given class, while recall (sensitivity) is the fraction between correct predictions for a given class and the total of cases in which it occurs. Accuracy reached a value maximum of 80%, while the highest value of precision was 95% in the third class. This high performance of the last class proves to be a strategic note because it allows us to derive more information on the most critical conditions characterized by prolonged LOS. The ROC curves for DT are reported in Figure 5.

**Figure 6:**
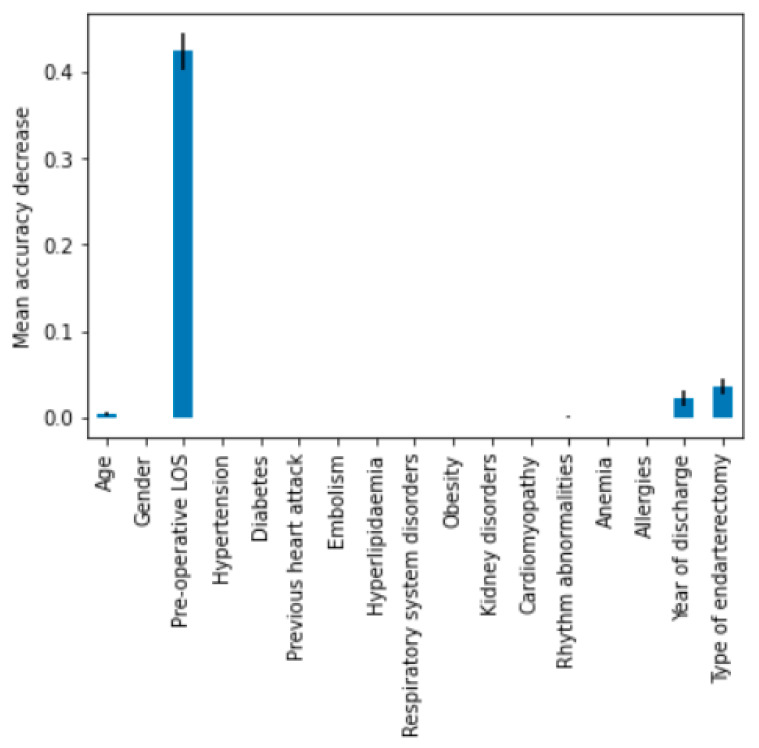
Result of permutation feature importance.

The macro-average ROC curve area was equal to 0.85. Lastly, feature importance permutation was implemented to assess which independent variables most influenced the model. This procedure effectively breaks the link that exists between one of the independent variables and the dependent variable in order to identify how much the model depends on that particular feature. The importance of a feature was determined by evaluating how a model’s reference score (such as accuracy) changes using a corrupted version of the data on that specific variable. Figure 6 shows the importance ranking for DT.

As expected, pre-operative LOS was the variable that significantly affected total LOS, followed by type of endarterectomy and year of discharge. Combining the estimators together, using a ‘hard’ voting technique, an accuracy of 79% was obtained. Through a majority vote, a slightly lower accuracy was produced than the vote obtained from DT alone, which can be attributed to the fact that more classifiers misclassify the same instance than DT. Using the weights determined based on the level of accuracy, the model improves from a value of 0.795 to 0.797 approaching the expected value.

**Figure 7:**
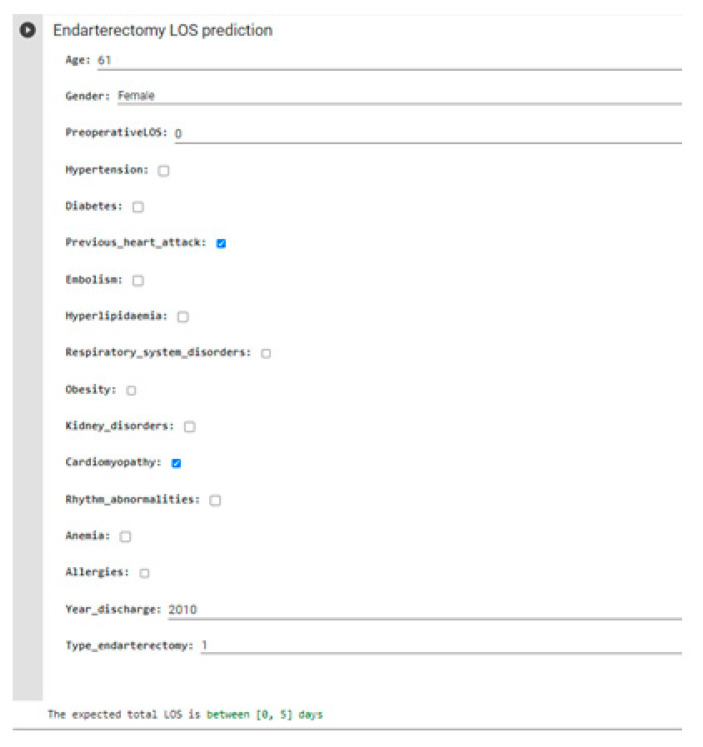
Module created for manual input of input data that returns the prediction of total LOS as output.

Having identified DT with the best classifier, it is possible to create a form on Google Colab with which healthcare personnel can enter input parameters and obtain the predicted total LOS. Figure 7 shows an example of a real case with a total LOS of 4 days.

## 4. Discussion

Endarterectomy is a high-efficiency surgical treatment for stroke prevention that is becoming increasingly popular. Inpatient costs, on the other hand, are rising at a similar rate. Because LOS is such an important factor in the cost of endarterectomy, predicting this metric for patients can be a useful tool to prioritize quality improvement efforts and prepare sufficient resources.

In this study, the data of 2243 patients who underwent endarterectomy were collected at the Complex Operative Unit (C.O.U.) of Cardiology of the “San Giovanni di Dio e Ruggi D’Aragona” University Hospital of Salerno (Italy). Three different regression models and five different classification algorithms were performed to predict LOS considering different inputs, i.e., gender, age, hypertension, diabetes, previous heart attack, embolism, occlusion or stenosis, atherosclerosis, hyperlipidaemia, respiratory system disorders, obesity, kidney disorders, cardiomyopathy, rhythm abnormalities, anemia, allergies, pre-operative LOS, type of endarterectomy, and then compered their evaluation metrics.

Compared with the short paper, which analysed a subset of this dataset with a reduced number of variables, RF was not confirmed as the best algorithm, achieving generally lower accuracy performance [34]. The use of ML to predict total in-hospital LOS for medical patients has been assessed by several studies with different methodologies and results [36]. Examining the variables that influence LOS, Rodd et al. [26] show that diabetes mellitus is also a predictive factor in their study. In contrast to our study, Hernandez et al. [22] show how female sex is associated with prolonged LOS, but both highlight how there is no relationship with factors such as myocardial infarction or atrial fibrillation. Scala et al. [37], on the other hand, demonstrate how preoperative hospital stay is a strong predictor, partly because it is included in total LOS by definition. The type of endarterectomy, as evidenced by our study, also significantly influences LOS. Pollard et al. [38] mainly show the impact that a preliminary outpatient evaluation can have on preoperative LOS, but they also show the substantial differences between the lower extremity and carotid procedures. Finally, renal diseases, especially chronic renal failure, have been the subject of several studies. Sidawy et al. [39] highlight the importance, for these patients, of careful preoperative screening for possible cardiologic or pulmonary complications that could significantly affect LOS.

In the field of cardiology, several studies have been conducted to investigate the impact of patients’ clinical and demographic variables on LOS [40]. For example, Daghistani et al. [41] developed an application very similar to that discussed in this study, including several cardiology procedures in a smaller number of years of observation and analysis models. Other studies, however, are limited to the use of statistical analysis [19] or exclusively regression models [42] although on a larger number of clinical variables using medical records as a source. In addition, the use of alternative methods, such as carotid artery stenting (CAS), not considered here, is analysed. CAS is a less invasive procedure conducted under local anesthesia, less affected by the comorbidities of the patient, who is usually discharged the next day [43]. Randomized studies have shown that CAS has a slightly higher cost, however is acceptable by cost-effectiveness standards, and is associated with a higher risk of periprocedural stroke or death than endoarterectomy. This additional risk is related to an increase in nondisabling strokes occurring in people older than 70 years [44,45].

The strength of this study is that it is a large-scale analysis involving a considerable number of years of observation and variables, being able to compare the results of different ML algorithms. The high performance of classification models on the class with prolonged LOS demonstrates the benefits that a healthcare facility can gain from this type of implementation. The clinical implications are related not only to a field implementation of the models that could lead to more agile healthcare programming and planning, but also for a more in-depth study of the procedure under investigation. Identifying which variables most influence LOS could help healthcare management to identify possible risk factors or for the identification of protocols to be adopted on specific categories of patients.

The limitations of this work, as already anticipated, are mainly related to the source of the data. It was not possible to include clinical factors or to characterize in detail the degree of complexity of the diseases considered. In addition, the impact on the total LOS of any other procedures delivered during the same hospitalization and the effects caused by the COVID-19 pandemic were not considered. Finally, it should be pointed out that although our results are in line with what can be found in the literature, the fact that it is a single-center study limits the generalization of results, which could depend on factors related to the organization of the hospital and the surgeons performing the procedure.

## 5. Conclusions

Hospitals are significantly reducing costs, while public health spending in Italy has increased significantly. The length of stay (LOS) is a major factor in calculating public expenditure. In recent years, LOS after endarterectomy has increased significantly, causing an increase in public spending. For this reason, the present study was conducted to predict LOS using different machine learning algorithms. By comparing these algorithms, the one that can most accurately predict LOS can be identified.

Future directions include expanded data collection to increase the number of predictors and the overall dataset size in order to achieve a more accurate and efficient prediction model.

## Figures and Tables

**Table 1:** Discharge distribution of patients undergoing endarterectomy included in the study by year.

Year of Discharge	2010	2011	2012	2013	2014	2015	2016	2017	2018	2019	2020
N° of discharges	60	286	252	222	246	222	215	196	185	208	151

**Table 2:** Distribution of the number of discharges for endarterectomy patients included in the study according to the type of endarterectomy.

Type of endarterectomy	1	2	3	4
N° of discharges	2097	4	3	139

**Table 3:** Selected values of each hyperparameter for the chosen ML algorithms.

Algorithms	Hyperparameters
SVM	‘kernel’: (‘linear’, ‘rbf’), ‘C’: [1, 10, 100], cv = 10
RF	‘n_estimators’: [5, 10, 15, 20], ‘max_depth’: [2, 5, 7, 9], cv = 10
DT	‘max_depth’: range(3, 20), cv = 10
MLP	‘hidden_layer_sizes’: [(50, 50, 50), (50, 100, 50), (100,)], ‘activation’: [‘tanh’, ‘relu’], ‘solver’: [‘sgd’, ‘adam’], ‘alpha’: [0.0001, 0.05],’ learning_rate’: [‘constant’,’adaptive’], cv = 10
NB	‘var_smoothing’: np.logspace(0, −9, num = 100), cv = 10

**Table 4:** Results of regression analysis. R-squared, R-squared adjusted and root mean square error (RMSE) are reported for each model.

	MLR	RF	DT
**R-squared**	0.845	0.782	0.584
**R-squared adjusted**	0.840	0.775	0.571
**RMSE**	2.217	2.628	3.630

**Table 5:** MLR parameters: regression coefficients, *t*-test and *p*-value.

	Unstandardized Coefficients		Standardized Coefficients	t	*p*−Value *
	B	Std. Error	Beta		
Intercept	17.663	38,936	−	0.454	0.650
Age	0.007	0.007	0.012	1.030	0.303
Gender	0.063	0.116	0.006	0.539	0.590
Pre−operative LOS	1.013	0.015	0.781	66.633	**0.000**
Hypertension	−0.003	0.113	0.000	−0.029	0.977
Diabetes	0.348	0.117	0.034	2981	**0.003**
Previous heart attack	0.069	0.184	0.006	0.377	0.707
Embolism	0.214	0.407	0.007	0.527	0.598
Hyperlipidaemia	−0.095	0.113	−0.010	−0.847	0.397
Respiratory system disorders	0.071	0.117	0.007	0.607	0.544
Obesity	−0.023	0.364	−0.001	−0.062	0.950
Kidney disorders	0.515	0.188	0.031	2.745	**0.006**
Cardiomyopathy	−0.119	0.151	−0.012	−0.789	0.430
Rhythm abnormalities	−0.231	0.218	−0.012	−1.062	0.288
Anemia	−0.189	0.426	−0.005	−0.444	0.657
Allergies	−0.060	0.241	−0.003	−0.250	0.803
Year of discharge	−0.008	0.019	−0.005	−0.403	0.687
Type of endarterectomy	1.146	0.094	0.174	12.152	**0.000**

* p-value is statistically significant as p *≤ 0.05.*

**Table 6:** Baseline characteristics of the three groups identified.

Variables	LOS			*p*-Value *
	Group 0 N = 652	Group 1 N = 805	Group 2 N = 786	
Age	71.8 ± 7.9	72.1 ± 8.0	71.8 ± 8.8	0.754
Gender				
0	414	523	536	0.152
1	238	282	250	
Pre-operative LOS	1.2 ± 0.6	2.8 ± 0.9	7.1 ± 0.2	**0.000**
Hypertension				
0	414	498	443	**0.013**
1	238	307	343	
Diabetes				
0	455	553	504	**0.046**
1	197	252	282	
Previous heart attack				
0	554	658	632	0.334
1	108	147	154	
Embolism				
0	645	797	739	**0.000**
1	7	8	47	
Hyperlipidaemia				
0	329	430	464	**0.004**
1	323	375	322	
Respiratory system disorders				
0	431	526	505	0.758
1	221	279	281	
Obesity				
0	631	789	772	0.151
1	21	16	14	
Kidney disorders				
0	602	731	700	0.103
1	50	74	86	
Cardiomyopathy				
0	440	545	506	0.302
1	212	260	280	
Rhythm abnormalities				
0	614	758	718	**0.041**
1	38	47	68	
Anemia				
0	638	792	776	0.429
1	14	13	10	
Allergies				
0	617	758	745	0.852
1	35	47	41	
Year of discharge				
2010	10	27	23	0.179
2011	87	103	96	
2012	72	106	74	
2013	59	83	81	
2014	60	84	102	
2015	74	72	76	
2016	59	78	78	
2017	63	67	66	
2018	55	59	71	
2019	58	81	69	
2020	55	46	50	
Type of endarterectomy				
1	643	789	665	**0.000**
2	3	1	0	
3	0	0	3	
4	6	15	118	

* *p*-value is statistically significant as *p* ≤ 0.05.

**Table 7:** Accuracy and best parameters of the selected ML algorithms.

Algorithms	Accuracy	Best Parameters
RF	0.77	‘max_depth’: 9, n_estimators’: 15
MLP	0.78	‘activation’: ‘tanh’, ‘alpha’: 0.05, ‘hidden_layer_sizes’: (100), ‘learning_rate’: ‘constant’, ‘solver’: ‘adam’
NB	0.73	‘var_smoothing’: 0.001
SVM	0.79	‘C’: 10, ‘kernel’: ‘linear’
DT	0.80	‘max_depth’: 5
VC	0.79	‘voting technique’: hard, ‘weights’: None

**Table 8:** Evaluation metrics (precision, recall and F1-score) for each class with the best algorithm DT.

Algorithms	Class	Precision	Recall	F1-Score
DT	0	0.78	0.82	0.80
	1	0.71	0.79	0.75
	2	0.95	0.78	0.86

## Data Availability

The datasets generated and/or analysed during the current study are not publicly available for privacy reasons but are available from the corresponding author on reasonable request.

## Abbreviations

LOS	length of stay
ML	machine learning
MLR	multiple linear regression
DT	decision tree
RF	random forest
SVM	support vector machine
MLP	multilayer perception
NB	naive Bayes
VC	voting classifier
